# The Agile Co-production and Evaluation framework for developing public health interventions, messaging and guidance

**DOI:** 10.3389/fpubh.2023.1094753

**Published:** 2023-06-26

**Authors:** Lucy Yardley, Sarah Denford, Atiya Kamal, Tom May, Jo M. Kesten, Clare E French, Dale Weston, G. James Rubin, Jeremy Horwood, Matthew Hickman, Richard Amlôt, Isabel Oliver

**Affiliations:** ^1^NIHR Health Protection Research Unit in Behavioural Science and Evaluation, University of Bristol, Bristol, United Kingdom; ^2^Population Health Sciences, Bristol Medical School, University of Bristol, Bristol, United Kingdom; ^3^School of Psychological Science, University of Bristol, Bristol, United Kingdom; ^4^School of Psychology, University of Southampton, Southampton, United Kingdom; ^5^NIHR Applied Research Collaboration West (NIHR ARC West), University Hospitals Bristol and Weston NHS Foundation Trust, Bristol, United Kingdom; ^6^School of Social Sciences, Birmingham City University, Birmingham, United Kingdom; ^7^UK Health Security Agency, London, United Kingdom; ^8^NIHR Health Protection Research Unit in Emergency Preparedness and Response, King's College London, London, United Kingdom

**Keywords:** public health, interventions, co-production, evaluation, emergency response

## Abstract

A lesson identified from the COVID-19 pandemic is that we need to extend existing best practice for intervention development. In particular, we need to integrate (a) state-of-the-art methods of rapidly coproducing public health interventions and messaging to support all population groups to protect themselves and their communities with (b) methods of rapidly evaluating co-produced interventions to determine which are acceptable and effective. This paper describes the Agile Co-production and Evaluation (ACE) framework, which is intended to provide a focus for investigating new ways of rapidly developing effective interventions and messaging by combining co-production methods with large-scale testing and/or real-world evaluation. We briefly review some of the participatory, qualitative and quantitative methods that could potentially be combined and propose a research agenda to further develop, refine and validate packages of methods in a variety of public health contexts to determine which combinations are feasible, cost-effective and achieve the goal of improving health and reducing health inequalities.

## Introduction

We have learned from the COVID-19 pandemic that health protection would benefit from improved methods of rapidly co-producing, optimising and evaluating public health interventions, guidance and messaging (1, 2). This could ensure that all population groups receive practical and accessible interventions and messaging that help people protect themselves and their communities (3–5). Particular attention to how best to include and support people from diverse contexts and underserved communities is required (6, 7). Evidence for the effectiveness of specific interventions and public health advice aimed at facilitating behaviour change during the COVID-19 pandemic was limited (3, 8), highlighting the need for methods of rapid evaluation in order to determine what interventions do or do not successfully change behaviour and reduce infections, in which contexts, and why.

In this paper, we propose the Agile Co-production and Evaluation (ACE) framework for developing public health interventions and messaging. ACE combines three key ingredients necessary for effective and efficient public health intervention and message development: (i) speed, (ii) co-production with target communities and (iii) evaluation.

Whilst good progress has been made in successfully deploying many elements of the required methodologies in isolation, there is currently no comprehensive, cost-effective, validated framework for combining them. Guidance documents for developing interventions (9) and applying behavioural science to national policies do exist (10). However, existing evaluation frameworks do not combine all three of the components that comprise ACE. For example, excellent intervention development frameworks exist, but these have not addressed the needs of rapid development of public health interventions for emergencies (9, 11). Existing evaluation guides may lack detail about efficient co-design (10), and while there are established methods for inclusive co-design [e.g., (12)], these often depend on much longer timescales than are available when responding to public health emergencies. A review of the literature by members of the team revealed no comprehensive framework that combines rapid co-production with rapid evaluation currently exists (13). The need for such a framework is further supported by qualitative work conducted with behavioural scientists and public health practitioners during the COVID-19 pandemic. That work revealed that whilst frameworks for supporting the application of behavioural science into public health policy are useful, existing frameworks were considered insufficient for pandemic situations, and it was felt that a co-produced strategy would be helpful (14).

We suggest the ACE framework can provide a unifying focus and flexible agenda for a programme of methodological innovation to supplement existing best practice in intervention development by ensuring that rapidly co-produced interventions and messaging are appropriate for all target users and effective when implemented.

Below, we describe ACE and how it was developed. Drawing on our experience during the COVID-19 pandemic, we outline some examples of methods that could be integrated within the framework.

## Conception and development of the ACE framework

The ACE framework was conceived by a team of behavioural and social scientists, public health professionals and members of the public. Our collective struggles with rapidly developing and evaluating interventions during the COVID-19 pandemic convinced us that better approaches were urgently required. In particular, the speed at which interventions had to be developed and deployed meant that it had not always been possible to engage with those who required support the most, to rapidly evaluate co-produced interventions in terms of the impact on attitudes, acceptability and behaviour, or to rapidly evaluate the implementation of interventions in the real world.

During a series of meetings, we characterised the core challenges we had faced and discussed research agendas and strategies that could be developed ahead of future emergency situations to support effective intervention development and evaluation. The ACE framework was proposed by the lead author and discussed and refined through consultation with the core team. Following this, the team conducted a scoping review that aimed to map available behavioural science resources that could be used to develop and evaluate public health guidance, messaging, and interventions in emergency contexts onto components of ACE. Of the 17 studies that were included in the review, three discussed co-production with the target audience and consideration of diverse populations, four focused on rapid testing, evaluation, or validation methods, and six were designed to support rapid implementation. None included all components of ACE. This confirmed the need for such a framework, and a paper in which a prototype framework was described was co-developed and circulated among the team for an interactive discussion.

As an initial test of our original framework, which focused primarily on the co-production of public health messaging, we applied it to the development of messages to support members of the public to protect themselves from mpox (formerly monkeypox) (15). Mpox almost exclusively affected sexual networks of gay, bisexual or other men who have sex with men (GBMSM) and people living with HIV and was declared a public health emergency of international concern by the World Health Organization (WHO) on July 23rd 2022. In efforts to control transmission, multiple public health measures were introduced, including vaccination, contact tracing and isolation. There was a need for rapid research exploring facilitators for and barriers to the uptake of public health measures among GBMSM to inform optimizations of the intervention measures. This first application of the framework quickly revealed the need for substantial modifications to the framework. In particular, we became aware of the need to focus not just on public health messaging, but on complex interventions to support behavior change.

At this point, we approached public contributors and invited them to share their perspectives. These contributors comprised 3 women and 2 men (1 Black British, 1 Mixed Black Caribbean and White, 2 British Pakistani, 1 White) who worked in community, health, or public health settings during the COVID-19 pandemic. They reviewed a draft of this paper and gave their views on how compatible the ACE framework is with real-world experiences, and opportunities and challenges for this approach. Verbal (MS Teams, telephone) and written feedback was obtained in English and Urdu between 12th and 21st September 2022. The feedback was very positive about the recommended approach and methods but suggested some changes to the text and advised that a Figure was needed to summarise the key elements of the ACE approach to aid understanding and implementation in practice. Numerous recommendations were also given for methods of co-production, and these were added to Table 1 or the text. The revised text and Figure were recirculated to the public contributors to check that their suggestions had been well represented in the paper.

**Table 1:** Potential methods for applying the ACE framework – to be expanded, refined and validated through use and practice.

PPI and stakeholder involvement at all stages of intervention and messaging co-production, implementation and evaluation as full members of the research team, including: members of the public, especially a range of people from seldom-heard groups and communities; healthcare professionals and providers, including people from diverse backgrounds; community leaders; behaviour change experts and relevant others. Involvement in co-production teams of people with cultural sensitivity and competency, a deep understanding of inequalities, and good listening and communication skills, including in languages used by the target communities.
Capacity building and consultation with local communities to support engagement with co-production while avoiding community members being over-burdened or feeling excessively targeted. This should involve working with trusted community influencers but also listening to a wide range of people with different views. Real-time analysis of linked data to identify the sectors of the population that are underserved and/or have high need. Co-production of interventions and messages that reflect the priorities of the target groups and communities as well as public health priorities, taking a holistic approach to wellbeing. Co-production of interventions and messaging tailored and targeted appropriately for specific (e.g., population or geographic) context, using suitable language, messengers, imagery and delivery formats (e.g., social media, flyers, radio). Rapid iterative adaptation and optimisation of interventions and messages based on evaluation findings to ensure that they are accessible, feasible and evidence-based and are easily understood and seen as appropriate, relevant and useful by target members of the population.
Rapid online surveys to determine knowledge and perceptions of disease/infection control behaviours among the general population. Rapid surveys administered in person at appropriate locations (e.g., school, work, hospital, community centre) to include members of the population less likely to engage with online surveys (e.g., who do not have digital access or prefer in person communication with known and trusted people). Large-scale in person consultations (with facilitators from diverse groups that have in-depth knowledge of cultural competency) using pre-established national and local networks to include people who are less likely to engage with online consultations.
Large online experiments to test effects of co-produced public health messages/interventions on intentions to engage in infection control behaviour(s).
Semi-automated analysis of large free text data-sets: use of artificial intelligence methods to assist qualitative researchers to analyse responses to open-ended survey questions from large population samples.
Qualitative and quantitative analysis of comments, posts and features shared *via* social media, including audio messages, to understand views and attitudes of members of the public who do not wish to take part in surveys or interviews.
Rapid participatory ethnographic research, with appropriately skilled community members carrying out observation, interviews and surveys.
Audit of feasibility and implementation of interventions to determine if specific, measurable, timely actions have been undertaken by those involved in implementing them. Rapid evaluations using routine data to assess outcomes in a range of real-world implementation settings using observation, experimental or quasi-experimental methods.
Mixed methods process evaluations: rapid qualitative and quantitative research aiming to understand system effects and impacts on objective measures of behaviour and health.

## Overview of the ACE framework

**Figure 1:**
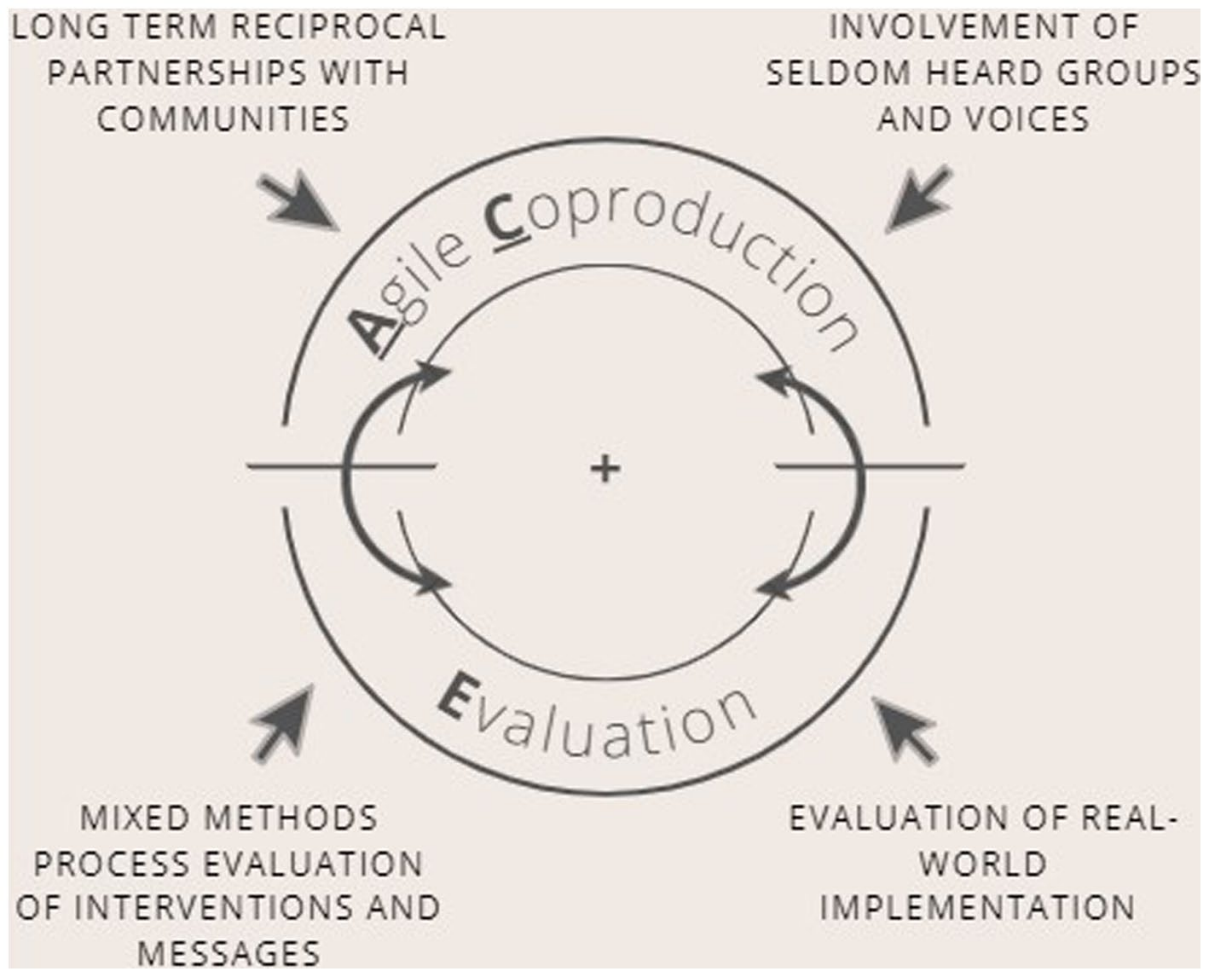
Overview of the Agile Co-production and Evaluation (ACE) Framework.

The ACE framework is an intervention development and evaluation framework and describes the process by which interventions may be rapidly co-produced and evaluated so that they reach all the target users, and meet the priorities of the public, particularly those from seldom-heard communities. The framework combines the three key ingredients (i) an agile approach to intervention development, (ii) co-production with target communities and (iii) evaluation (Figure 1) which we define below.

Agile: Agile intervention development is a concept that originated in the field of software development but can be applied to healthcare intervention development (16). Agile development typically involves a rapid cycle of user-centred development in which evaluation of the user experience informs rapid optimisation. Public health interventions frequently need to be developed, evaluated, and implemented very quickly. Thus agile methods are required to speed up the co-production and evaluation cycle. For example, this may include making use of pre-established or existing systems and relationships to facilitate the conduct of rapid recruitment, engagement, and/or data collection, together with rapid analysis methods.

Co-production: Co-production involves researchers, practitioners and members of the public working together to achieve a shared outcome (17). The term co-production often means different things to different people, in part due to the wide range of disciplines from which co-production originates (18). We use the term inclusively to reflect the range of definitions and ways of working; in recognition of the need for flexibility in how co-production is used and achieved, depending on the context, situation and target audience.

Evaluation: Finally, we use the term “evaluation” to include efforts to assess the acceptability and feasibility of the intervention, factors influencing effective implementation, the process by which the intervention leads to impact, and/or impact of interventions through rapid testing, evaluation or other validation methods (such as online testing or implementation evaluation using routine data).

ACE is intended to be used flexibly to suit the needs of the specific context in which the intervention is being developed. Figure one depicts the cyclical nature of the framework, indicating that the framework should not be considered a linear process but may be applied at any point in the cycle of developing, adapting, optimising or implementing an intervention.

Below we suggest some methods and recommendations that may be usefully employed during each of the components.

## Agile co-production and optimisation of appropriately targeted and tailored interventions and messaging

Co-production is vital to ensure that both population-level and targeted interventions and messaging are appropriate for and trusted by the people and communities that they must engage (10, 19, 20). It is crucial that co-production involves communities or groups whose voices are seldom heard from during emergency public health campaigns and who may face additional barriers, such as those from lower socio-economic groups and ethnically diverse communities (21). This may require innovative approaches to engage with members of these groups, including offering a wider variety of ways in which members of the public can contribute and establishing new forms of long-term partnerships (22). Long-term reciprocal relationships established prior to emergencies can not only build trust and help to break down ‘them/us’ barriers but can then facilitate rapid co-production in emergencies. Offering a wide range of digital and non-digital methods (written, oral/aural, visual and in-person) for involving and communicating with members of the public is necessary to meet the needs and preferences of seldom heard members of the population, including people of all ages and with disabilities.

Many public health teams have excellent ongoing partnership links with their diverse communities and the skills to co-produce interventions and messaging in consultation with different groups (6, 7). The UK Community Champion schemes are an example of a successful innovative and responsive approach to increasing engagement with diverse communities during the COVID-19 pandemic. The schemes provided a supportive framework for building capacity in seldom-heard communities to generate rapid insights and co-create interventions and messages that reflected the needs and attitudes of specific groups (23).

Targeting and tailoring of interventions and messages will benefit from being informed by the best available evidence wherever possible. For example, during the COVID-19 pandemic target user groups were identified using real-time analysis of linked data to detect and include the sectors of the population that were underserved and had high need for targeted interventions such as vaccination outreach (19). Other examples include use of community-led researchers and local resilience forums to provide real-time qualitative data to inform the development of targeted interventions (24).

Once interventions and messages have been co-produced, evaluation of their effectiveness in achieving intended outcomes is required. However, to date, public health and research teams have not usually combined their co-production efforts with experimental testing or objective validation. The aim of the ACE framework is to combine rapid intervention and messaging co-production with immediate evaluation and further optimisation. This could include some of the approaches outlined below.

## Rapid mixed methods evaluation of interventions and messages

In the COVID-19 pandemic, rapid large-scale online surveys were used to establish attitudes and intentions towards planned or ongoing interventions, such as testing, self-isolation and vaccination (25, 26). Experimental online evaluation (such as A:B testing) of the likely impact of messages on attitudes and intentions was used to test their relative effectiveness and to modify the messages accordingly (27–29). For example, an online experiment found that adding a single sentence informing participants that there was still a chance that they could be infectious significantly improved participant understanding of the risk of transmitting COVID-19 following a negative test result (30). Online studies evaluating perceived importance of mitigation measures among individuals attending cultural events informed implementation of infection control polices at mass events (31).

Online message testing has significant limitations – it can only tap into self-reported hypothetical intentions among people able and willing to take part in online studies, which will exclude important sectors of the population even when representative sampling is employed. However, as part of an evaluation package this method can provide useful evidence concerning the relative effects of different messages on attitudes and intentions (usually a necessary albeit not sufficient precursor of behaviour) in a large population sample. This method could be used to screen out messages with less potential for positive impact, compare the effects of different messages and identify messages that may have differential impact on different sectors of the population. In future, it may be possible to conduct rapid large-scale evaluations that are not online by creating the required infrastructure of well networked national, local and grassroots community groups that can be called on as required.

The value of large-scale testing is likely to be substantially enhanced if it is combined with the co-production element of the ACE package, which should ensure that the messages that are tested online have the greatest potential to be acceptable, credible and effective in different communities. Rapid analysis of large-scale qualitative datasets, such as social media, can also usefully supplement large-scale surveys. For example, semi-automated methods of qualitative analysis could permit use of open-ended questions about the reasoning behind survey responses (32).

An essential element of evaluating interventions and messaging is to measure their real-world impact on behaviour and health outcomes. In an emergency this will need to be carried out immediately after the intervention is implemented, to inform ongoing management of the emergency. For example, rapid studies were carried out during the COVID-19 pandemic to evaluate interventions and messaging to try to reduce transmission in large venues (33–35). It is vital to develop methods of objectively measuring outcomes, since the pandemic demonstrated that real world effects are often different from those predicted or anticipated, and reported attitudes, intentions and behaviour did not always statistically correlate highly with observed behaviour (36).

Rapid, pragmatic, low-cost methods of evaluation need to be developed in order to test and optimise interventions in a timely and cost-effective manner (37). Making use of existing or routinely collected behavioural and health data where possible could provide a feasible and pragmatic solution. Evolving learning health and care systems (38), should be able to provide the required infrastructure to carry out experimental or quasi-experimental efficient design implementation trials, using routine data to evaluate impacts on objective measures of behaviour and health, plus mixed methods process evaluations to understand system effects. As with all methods included in ACE, co-production of the evaluation with stakeholders will play a crucial role in identifying populations with the potential to benefit, appropriate methods of implementation and suitable outcome measures, and informing interpretation of process analyses of system effects (39).

## Framework refinement

The ACE framework has the potential to support the systematic development of effective, inclusive, and timely public health interventions. However, our discussion of potentially useful methods is far from exhaustive, and different methods will be appropriate in different intervention development contexts. The ACE research agenda now needs to further develop, refine and validate packages of methods in a variety of applications. Planned future work will involve validating the framework by applying it to a range of different health challenges, interventions and populations. In addition to this, we invite researchers to engage in discussion with us to collaboratively refine and optimise the framework so that it addresses the needs and challenges faced by others tasked with developing interventions in a time-pressured environment.

## Conclusion

In the COVID-19 pandemic there were some good examples of approaches to intervention and message development and evaluation that used co-production, large-scale experimental testing or were evaluated using objective measures (26) – but these methods were rarely combined and were not applied systematically. The ACE framework is intended to provide a focus for exploring a range of new ways of rapidly developing effective interventions and messaging by integrating co-production methods with experimental, quasi-experimental and real-world evaluation to secure better health outcomes. The ACE research agenda needs to further develop, refine and validate packages of methods in a variety of applications to determine which combinations of methods are feasible and cost-effective. If the ACE framework proves useful it could be applied for efficiently developing effective and timely public health interventions and messaging to facilitate adoption, maximise health benefit and reduce health inequalities in a range of contexts, including, importantly, the next public health emergency.

## Data availability statement

The original contributions presented in the study are included in the article/supplementary material, further inquiries can be directed to the corresponding author.

## Author contributions

All authors listed have made a substantial, direct, and intellectual contribution to the work and approved it for publication.

## Funding

This study was funded by the National Institute for Health and Care Research Health Protection Research Units (NIHR HPRU) in Emergency Preparedness and Response, a partnership between UKHSA, King’s College London and the University of East Anglia, and Behavioural Science and Evaluations, a partnership between UKHSA and the University of Bristol. LY, JK, and JH are partly funded by National Institute for Health and Care Research Applied Research Collaboration West (NIHR ARC West) and NIHR HPRU in Behavioural Science and Evaluation. For the purpose of open access, the author has applied a CC BY public copyright licence to any Author Accepted Manuscript version arising from this submission.

## Conflict of interest

The authors declare that the research was conducted in the absence of any commercial or financial relationships that could be construed as a potential conflict of interest.

## Publisher’s note

All claims expressed in this article are solely those of the authors and do not necessarily represent those of their affiliated organizations, or those of the publisher, the editors and the reviewers. Any product that may be evaluated in this article, or claim that may be made by its manufacturer, is not guaranteed or endorsed by the publisher.

## Author disclaimer

The views expressed are those of the author(s) and not necessarily those of the NIHR, Public Health England or the Department of Health and Social Care.
